# The Mechanism of Microcrack Initiation in Fe-C Alloy Under Tensile Deformation in Molecular Dynamics Simulation

**DOI:** 10.3390/ma18163865

**Published:** 2025-08-18

**Authors:** Yanan Zeng, Xiangkan Miao, Yajun Wang, Yukang Yuan, Bingbing Ge, Lanjie Li, Kanghua Wu, Junguo Li, Yitong Wang

**Affiliations:** 1 School of Metallurgy and Energy Engineering, North China University of Science and Technology, Tangshan 063210, China; zengyanann@126.com (Y.Z.); miaoxiangkan@stu.ncst.edu.cn (X.M.); wangyj@ncst.edu.cn (Y.W.); 15835683445@163.com (Y.Y.); 13131865718@163.com (B.G.); 2 HBIS Material Technology Research Institute, Shijiazhuang 050023, China; lilanjie20040014@163.com; 3 Nuclear Power Institute of China, Chengdu 610213, China; 18646085676@163.com

**Keywords:** molecular dynamics simulation, Fe-C alloy, temperature, void nucleation and growth, crystal structure, microcrack initiation

## Abstract

The microcrack initiation and evolution behavior of Fe-C alloy under uniaxial tensile loading are investigated using molecular dynamics (MD) simulations. The model is stretched along the *z*-axis at a strain rate of 2 × 10^9^ s^−1^ and temperatures ranging from 300 to 1100 K, aiming to elucidate the microscopic deformation mechanisms during crack evolution under varying thermal conditions. The results indicate that the yield strength of Fe-C alloy decreases with a rising temperature, accompanied by a 25.2% reduction in peak stress. Within the temperature range of 300–700 K, stress–strain curves exhibit a dual-peak trend: the first peak arises from stress-induced transformations in the internal crystal structure, while the second peak corresponds to void nucleation and growth. At 900–1100 K, stress curves display a single-peak pattern, followed by rapid stress decline due to accelerated void coalescence. Structural evolution analysis reveals sequential phase transitions: initial BCC-to-FCC and -HCP transformations occur during deformation, followed by reversion to BCC and unidentified structures post-crack formation. Elevated temperatures enhance atomic mobility, increasing the proportion of disordered/unknown structures and accelerating material failure. Higher temperatures promote faster potential energy equilibration, primarily through accelerated void growth, which drives rapid energy dissipation.

## 1. Introduction

The Fe-C alloy system, serving as the fundamental constituent of steel materials, has become indispensable in modern engineering due to its exceptional mechanical properties, wear resistance, and corrosion tolerance, with broad applications spanning the construction, automotive, and aerospace industries [1,2,3]. Nevertheless, enhancing the mechanical performance and service longevity of steel under extreme conditions—particularly under high-temperature and high-impact loading—remains a critical challenge in materials science. Traditional investigations into the dynamic mechanical behavior of steels have predominantly focused on macroscopic-scale experimental characterizations at varying temperatures and strain rates [4,5,6]. However, emerging evidence highlights that macroscopic properties are intrinsically governed by microstructural evolution, including crystallographic phase transitions [7,8], dislocation dynamics [9,10], stacking fault formation [11,12], twinning mechanisms [13,14], and void nucleation/growth processes [15,16,17], all of which play pivotal roles in determining material performance under dynamic loading.

Molecular dynamics (MD) simulations have emerged as a powerful tool for probing deformation mechanisms and fracture behaviors at atomic resolution, providing crucial insights into phenomena inaccessible to conventional experimental techniques [18,19]. Recent studies employing MD methodologies have systematically investigated microstructural responses to external stimuli in metallic systems. For instance, Wu et al. [20] revealed grain boundary sliding as the dominant deformation mechanism in polycrystalline iron at elevated temperatures. Wei et al. [21] correlated temperature variations with dislocation density evolution in copper. Investigations by Ding et al. [22] further elucidated the coupled effects of specimen dimensions, crack geometry, and strain rates on fracture propagation in aluminum. Notably, compositional modifications—such as carbon doping in Fe-C alloy—have been shown to induce strength variations depending on the atomic configuration [23]. Zhou et al. [24] extended these analyses to Fe-C systems, identifying enhanced mechanical performance at reduced strain rates and elevated temperatures. Jiao et al. [25] employed molecular dynamics simulations to investigate the deformation behavior of Fe-C alloys at high strain rates, revealing that phase transformation mechanisms and dislocation nucleation mechanisms differed depending on the carbon concentration. Yang et al. [26] discovered that different loading directions altered the distribution of C atoms within the Fe lattice and that specific arrangements of these C atoms induced BCC-to-HCP phase transformation. Wang et al. [27] investigated the effect of hydrogen on crack tip plastic deformation mechanisms in α-Fe using molecular dynamics simulations. Their study revealed that hydrogen deteriorates material plasticity and that plastic deformation mechanisms vary with the hydrogen concentration. Li et al. [28] investigated the effects of temperature and strain rate on the mechanical properties of Fe-C alloys. Their results demonstrated enhanced mechanical properties at high strain rates and low temperatures.

Above all, despite extensive research primarily focused on the mechanical properties and microstructural characteristics of Fe-C alloys at the microscale, the underlying mechanisms of microcrack initiation and evolution, as well as the influence of microstructural changes on microcrack formation, remain insufficiently understood and warrant further investigation. Therefore, this study employed molecular dynamics simulations to investigate the dynamic tensile behavior of Fe-C alloys across 300–1100 K. The effects of temperature on mechanical properties were systematically analyzed, the interplay between microstructural changes and crack initiation and evolution was elucidated, and the deformation mechanisms, as well as the initiation and propagation mechanisms of cracks, in Fe-C alloys under tensile loading were comprehensively revealed from a microscopic perspective.

## 2. Simulation Method

**Figure 1:**
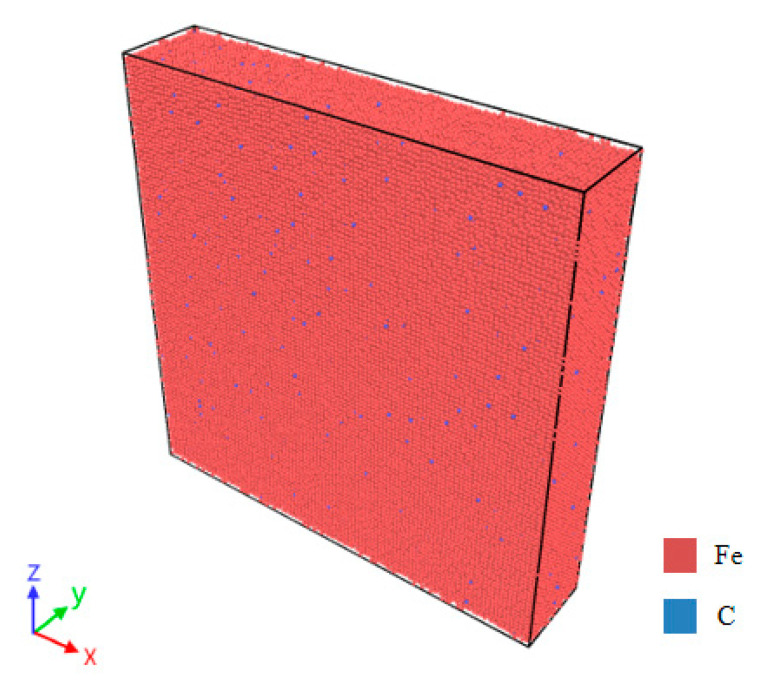
Simulation model of Fe-C alloy.

To elucidate the initiation and evolution mechanisms of microcracks at the atomic scale, the tensile process of Fe-C alloy within the temperature range of 300 K to 1100 K was simulated using LAMMPS [29]. A molecular dynamics model containing 400,000 bcc-Fe atoms was constructed with a lattice constant of 2.855 Å. The simulation cell dimensions were 28.55 nm × 5.71 nm × 28.55 nm, and the three axes X, Y, and Z corresponded to the crystal orientation of [100], [010], and [001], respectively. Carbon atoms were then randomly substituted for iron atoms at specific lattice sites to achieve a carbon concentration of 0.185 wt%, as illustrated in Figure 1. Following model construction, the system was equilibrated for 20 ps under the isothermal–isobaric (NPT) ensemble to ensure thermal equilibration at the target temperature prior to loading. Temperature regulation was maintained using the Nosé–Hoover thermostat, and energy minimization was conducted via the conjugate gradient (CG) method to achieve global energy convergence. Uniaxial tensile deformation was subsequently applied along the z-direction at a strain rate of 2 × 10^9^ s^−1^ under the canonical (NVT) ensemble. Ding et al. [19], Cao et al. [30], and Schiøtz et al. [31] pointed out that, due to the inherently limited time scale accessible through molecular dynamics simulations, the samples must undergo rapid deformation, and, in comparison with experimental studies, the process must be conducted under conditions of an extremely high strain rate. With the time step set to 1 fs, atomic-level stress, potential energy, and system volume along the loading direction were recorded at 100-step intervals throughout the simulation. The embedded atom method (EAM) potential [32] was employed to describe interatomic interactions within the Fe-C system.

Following the simulation, the results were subjected to post-processing and analysis using the visualization software Ovito version 3.13 [33]. The Common Neighbor Analysis (CNA) [34] and Dislocation Analysis (DXA) [35] methods were employed to identify local structural changes in the system during the deformation process. The CNA method assigns distinct colors based on the crystallographic structure: blue indicates a BCC structure, green indicates an FCC structure, red indicates an HCP structure, and white represents disordered atoms. The DXA algorithm identifies the location, type, and length of dislocations through the calculation of Burgers vectors. Additionally, a Python-based program was developed to quantitatively analyze the evolution of the void volume during the tensile deformation process.

## 3. Results and Discussion

In this section, the effects of varying temperatures on stress, total void volume fraction, dislocation density, crystal structure evolution, and potential energy during the tensile deformation of the system at a strain rate of 2 × 10^9^ s^−1^ are analyzed. Through a quantitative analysis of microstructural evolution, this study systematically elucidates the deformation mechanisms, as well as the initiation and evolution mechanisms of microcracks, during the tensile deformation of Fe-C alloys.

### 3.1. Stress–Strain Curve

**Figure 2:**
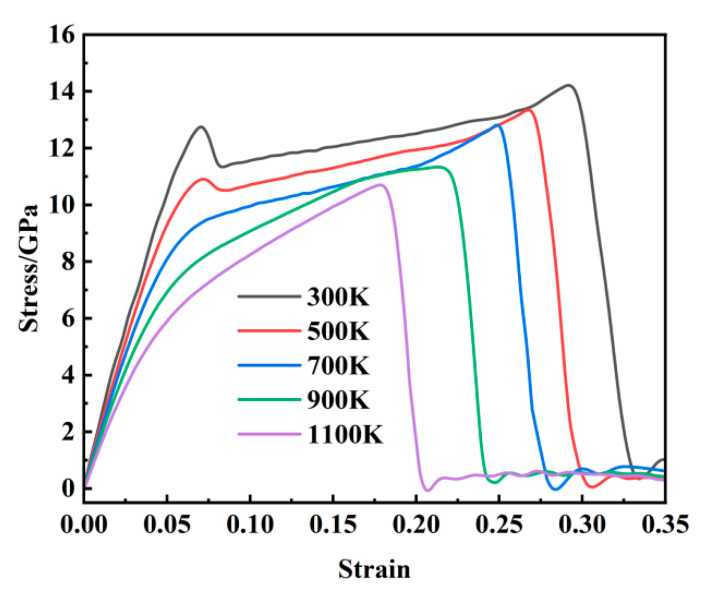
Stress–strain curves at 300–1100 K.

As illustrated in Figure 2, the stress–strain behavior of Fe-C alloy during tensile deformation exhibits distinct temperature-dependent characteristics across the temperature range of 300–1100 K. A double-peak pattern is observed at temperatures between 300 and 900 K, whereas a single-peak response is evident at 1100 K. Taking the 300 K case as a representative example, the stress–strain curve can be categorized into three distinct deformation stages for detailed analysis. The initial elastic stage is characterized by a stress–strain relationship that closely follows Hooke’s law, with an approximately linear stress increase up to the first peak of 12.23 GPa. The second stage corresponds to the yield regime, where a slight stress drop occurs immediately after the first peak, followed by a gradual and non-linear increase in stress until reaching the ultimate tensile strength of 14.31 GPa. This stage exhibits a significantly reduced strain-hardening rate compared to the elastic stage. The final stage represents plastic deformation, wherein the stress rapidly decreases with an increasing strain and subsequently stabilizes dynamically, indicating the initiation of fracture and the complete breakdown of interatomic bonding under applied loading.

**Figure 3:**
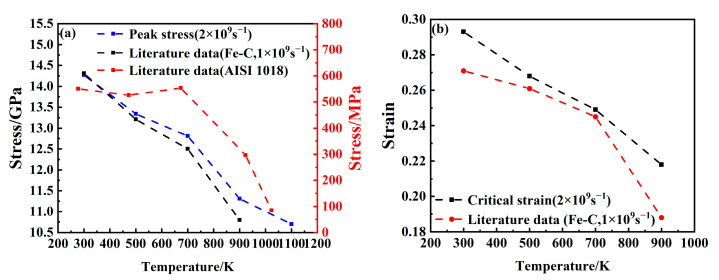
Comparison of mechanical property evolution of Fe-C alloy with literature data: (**a**) peak stress; (**b**) critical strain.

Figure 3a,b show a comparison of the mechanical property changes with the tensile processes of Fe-C alloy [28] and AISI 1018 steel [36]. By comparing them with the research results of Li et al. [28], it can be observed that both the stress peak and critical strain decrease progressively with an increasing temperature, with a more pronounced reduction at higher temperatures. Additionally, a double-peak feature in the stress–strain curve is identified in both this study and previous work. This is basically consistent with the rules obtained from the tensile tests of AISI 1018 steel at the macroscopic scale. The mechanical properties of the steel show an overall downward trend with an increasing temperature. As the temperature increases from 300 K to 1100 K, the stress peak declines from 14.27 GPa to 10.7 GPa, representing a reduction of 25.2%, while the critical strain decreases from 0.293 to 0.179. These observations indicate a significant influence of temperature on the mechanical behavior of the material. The observed reduction in mechanical strength is primarily attributed to the increased atomic kinetic energy at elevated temperatures, which enhances atomic vibration frequency and weakens interatomic bonding forces, ultimately reducing overall system stress.

### 3.2. Void Volume Fraction Analysis

**Figure 4:**
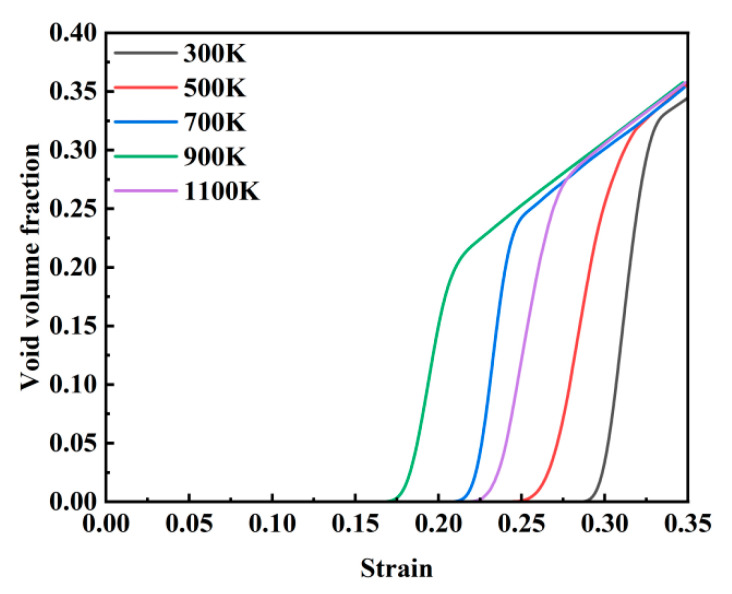
Evolution of void volume fraction with strain at 300–1100 K.

**Figure 5:**
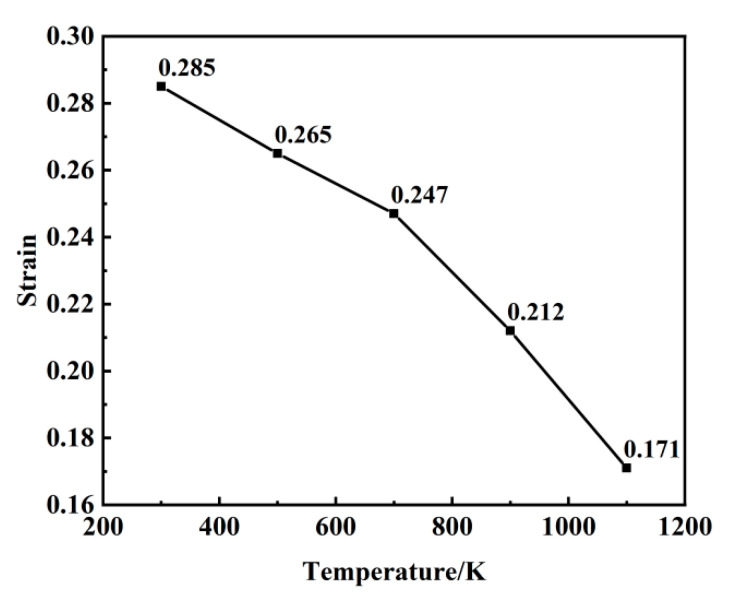
Evolution of void nucleation strain at 300–1100 K.

Figure 4 shows the evolution of the void volume fraction with strain across temperatures ranging from 300 K to 1100 K. While the stress–strain curves differ under varying thermal conditions, the void volume fraction exhibits consistent trends with an increasing strain. Higher temperatures significantly lower the critical strain required for void initiation, decreasing from 0.285 at 300 K to 0.171 at 1100 K. Notably, the final void saturation level stabilizes at approximately 35% across all temperatures. As detailed in Figure 5, temperature exerts a pronounced influence on void nucleation timing. The critical nucleation strains progressively decrease with rising temperatures: 0.171 at 1100 K, 0.212 at 900 K, 0.247 at 700 K, 0.265 at 500 K, and 0.285 at 300 K. This inverse correlation underscores the thermally accelerated nature of void nucleation. Elevated thermal energy enhances atomic mobility, enabling atoms to escape lattice sites more readily, thereby promoting premature void nucleation through localized bond rupture.

**Figure 6:**
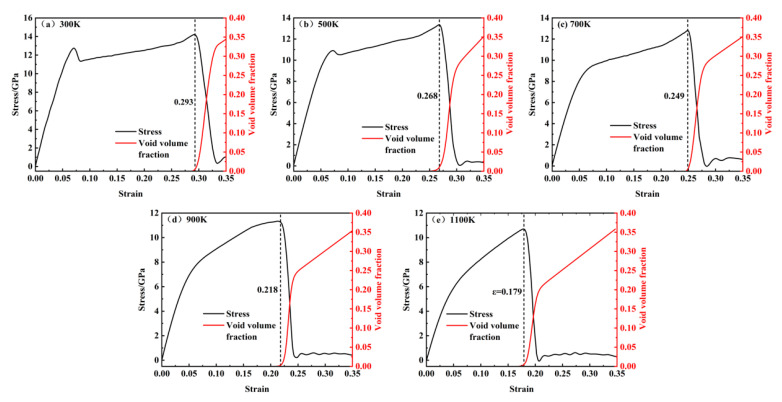
Relationship between void volume fraction and stress at 300–1100 K: (**a**) 300 K; (**b**) 500 K; (**c**) 700 K; (**d**) 900 K; (**e**) 1100 K.

Figure 6a–e show a comparison of the changes in porosity and stress with temperature ranging from 300 to 1100 K. The dashed lines in the figures correspond to the points near the maximum and minimum stress values. It can be observed that pore nucleation begins before the maximum stress point, with a slow increase in porosity. After reaching the maximum stress point, the rate of increase in porosity accelerates significantly. Therefore, the growth of porosity with temperature from 300 to 1100 K can be divided into three stages.

(1) Slow Growth Stage: Before reaching the maximum stress, pores begin to nucleate in localized areas and grow slowly. The porosity increases gradually, indicating that the large-scale growth of pores has not yet occurred. At this stage, the porosity is primarily due to pore nucleation. As temperature increases, pore nucleation occurs earlier; for instance, the strain required for pore nucleation at 300 K is 0.298, while at 1100 K, it decreases to 0.171, suggesting that pore nucleation is more likely at higher temperatures.

(2) Rapid Growth Stage: After the maximum stress is reached, pores grow rapidly, causing the porosity to increase rapidly. This stage exhibits the fastest rate of porosity increase, with the change approximately following an exponential growth pattern. By comparing this with the stress–strain curve, it can be seen that the rapid growth of porosity corresponds to the rapid decrease in stress, indicating that the rapid increase in porosity is the primary cause of the stress reduction in the system.

(3) Uniform Growth Stage: As most of the internal stress in the system is released, pores primarily aggregate and merge with neighboring pores. In this stage, the rate of increase in porosity slows down, and the growth trend becomes approximately linear. The total porosity at a strain of 0.35 reaches around 35% across different temperatures, with no significant difference, suggesting that temperature has little effect on the final porosity.

**Figure 7:**
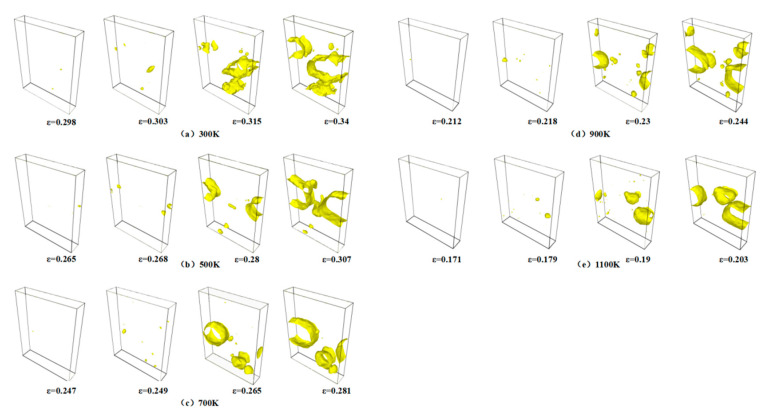
Distribution of voids at 300–1100 K: (**a**) 300 K; (**b**) 500 K; (**c**) 700 K; (**d**) 900 K; (**e**) 1100 K.

Material fracture primarily results from the nucleation, growth, and coalescence of voids at the microscopic level. Figure 7a–e illustrate the void distribution at four key deformation stages, namely, void nucleation, peak stress, stress drop, and minimum stress, respectively, capturing the evolution of void nucleation, growth, aggregation, and eventual coalescence under varying temperature conditions. The void density at the peak stress stage is notably higher at 1100 K than at 300 K, indicating more pronounced material damage at elevated temperatures. This phenomenon can be attributed to the enhanced atomic mobility induced by an increased temperature, which facilitates atomic displacement from equilibrium positions and promotes earlier and more extensive void nucleation. With further strain accumulation, the voids undergo growth and expansion, serving as the initial mechanism for microcrack initiation. The subsequent aggregation and coalescence of adjacent voids lead to progressive microcrack propagation and widening, ultimately resulting in macroscopic crack formation and structural failure.

### 3.3. Dislocation Density Analysis

**Figure 8:**
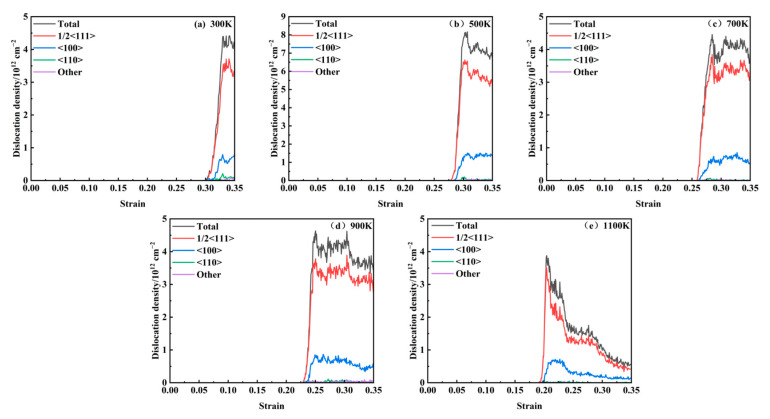
Evolution of dislocation density at 300–1100 K: (**a**) 300 K; (**b**) 500 K; (**c**) 700 K; (**d**) 900 K; (**e**) 1100 K.

**Figure 9:**
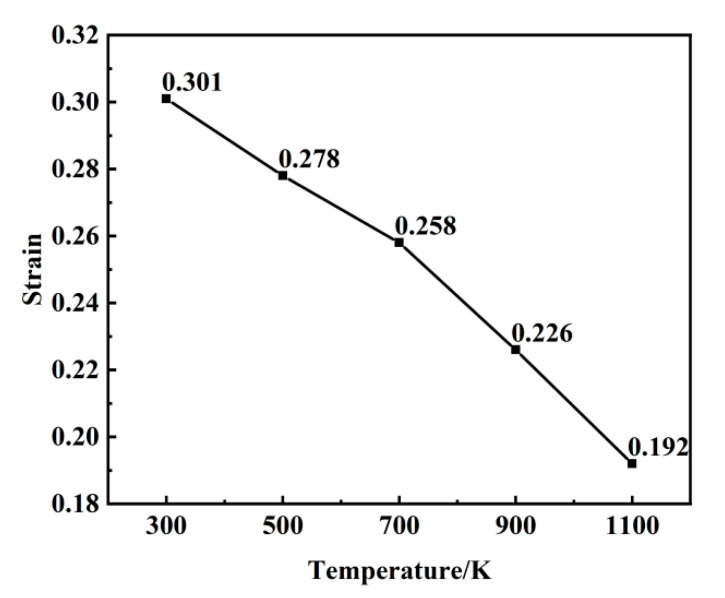
Dislocation nucleation points at 300–1100 K.

Figure 8 illustrates the correlation between dislocation density and strain across temperatures ranging from 300 to 1100 K. A comparative analysis with the stress–strain curve reveals that dislocations initiate after the peak stress and play a dominant role in the plastic deformation process. As observed in the data, dislocation nucleation begins following the stress maximum, and the dislocation density subsequently increases sharply with strain, reaches a peak, and then gradually declines. The primary dislocation types present during loading include 1/2<111>, <100>, and <110>. Dislocations that cannot be classified by the post-processing software are categorized as “Other” type, with the 1/2<111> dislocations being the most prevalent. As depicted in Figure 9, the strain values at which dislocations first appear at different temperatures are 0.31, 0.278, 0.258, 0.226, and 0.192. With an increasing temperature, the critical strain required for dislocation initiation decreases progressively. The generation and annihilation of dislocations constitute a dynamic process. During the early stages of plastic deformation, dislocations propagate rapidly, leading to a sharp increase in dislocation density. Once the density reaches its peak, dislocation interactions become more frequent, resulting in a gradual reduction in density. As temperature rises, thermal activation significantly enhances dislocation mobility, promoting more active dislocation generation and migration. This leads to intensified dislocation interactions and a more pronounced decrease in dislocation density. A comparable dislocation evolution mechanism was reported in the molecular dynamics simulations conducted by Zolnikov et al. [37].

**Figure 10:**
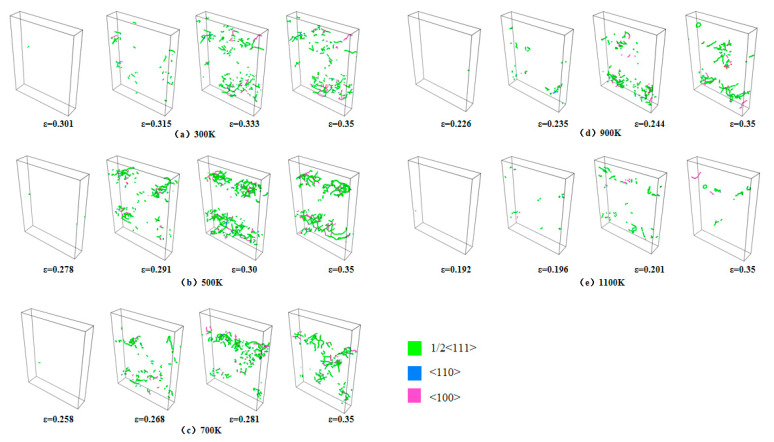
Distribution of dislocation at 300–1100 K: (**a**) 300K; (**b**) 500 K; (**c**) 700 K; (**d**) 900 K; (**e**) 1100 K.

Figure 10a–e depict the spatial distribution of dislocations at various strain stages across temperatures ranging from 300 to 1100 K. In these figures, green denotes 1/2<111> dislocations, blue denotes <100> dislocations, pink denotes <110> dislocations, and gray denotes unclassified dislocation types (“Other”). A comparative analysis with Figure 7 reveals that, upon reaching the peak applied stress, voids enter a rapid growth phase, leading to the formation of microcracks. Dislocations are predominantly located around these microcracks and exhibit slip behavior along <111> slip planes, thereby enhancing localized plastic deformation. With further strain accumulation, dislocations proliferate and migrate extensively, forming widespread dislocation structures. Localized dislocation pile-ups subsequently occur, inducing stress concentration zones that facilitate the rapid propagation of cracks. In the compression experiments carried out by Hagen et al. [38], 1/2<111> dislocations were identified, and these dislocations were found to play a key role in governing the plastic deformation behavior throughout the compression process.

### 3.4. Radial Distribution Function Analysis

**Figure 11:**
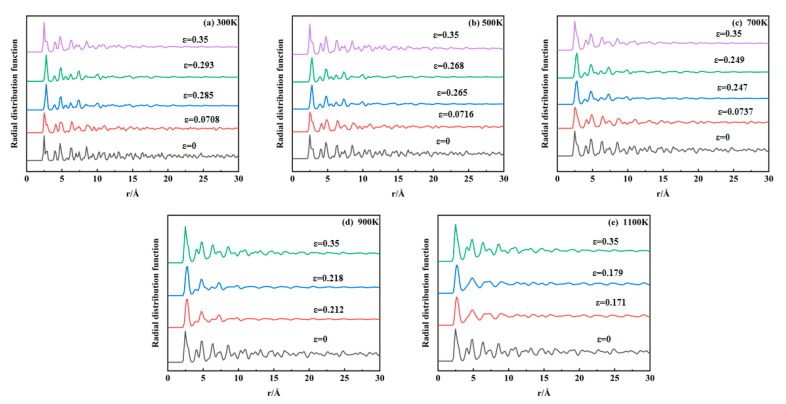
Evolution of radial distribution function at 300–1100 K: (**a**) 300K; (**b**) 500 K; (**c**) 700 K; (**d**) 900 K; (**e**) 1100 K.

The radial distribution function (RDF) describes the relative probability of finding another particle at a specific radial distance from a reference particle, serving as a key metric for analyzing atomic order or spatial arrangements within materials. In crystalline materials, periodic atomic arrangements yield long-range RDF peaks due to ordered lattice structures. Disruption of the crystallographic order eliminates these long-range features, enabling RDF-based detection of structural evolution during tensile deformation. Figure 11a–e display RDF profiles calculated using OVITO across 300–1100 K. For 300–700 K, five characteristic strain points are analyzed: initial tensile state, first stress peak, void nucleation onset, second stress peak, and near-stress minimum. For 900–1100 K, four points are selected: initial tensile state, void nucleation onset, stress peak, and near-stress minimum. At 300–700 K, the initial crystalline structure exhibits a long-range order characterized by persistent oscillations in the radial distribution function (RDF) profiles. Upon reaching the first stress peak, the long-range oscillations begin to attenuate. Following void nucleation, only short-range correlations remain significant, with distant oscillations collapsing into near linearity. Near the stress minimum, localized RDF fluctuations re-emerge, indicating partial structural reordering within the material. For 900–1100 K systems, the initial long-range order diminishes following void nucleation, demonstrating temperature-accelerated crystallographic disordering. Elevated temperatures broaden primary RDF peaks while reducing long-range oscillation amplitudes, reflecting thermally induced structural modifications. Notably, the RDF profiles remain nearly identical at stress peaks and void nucleation points, suggesting minimal structural impact from early-stage void nucleation due to low void volume fractions.

### 3.5. Common Neighbor Analysis

**Figure 12:**
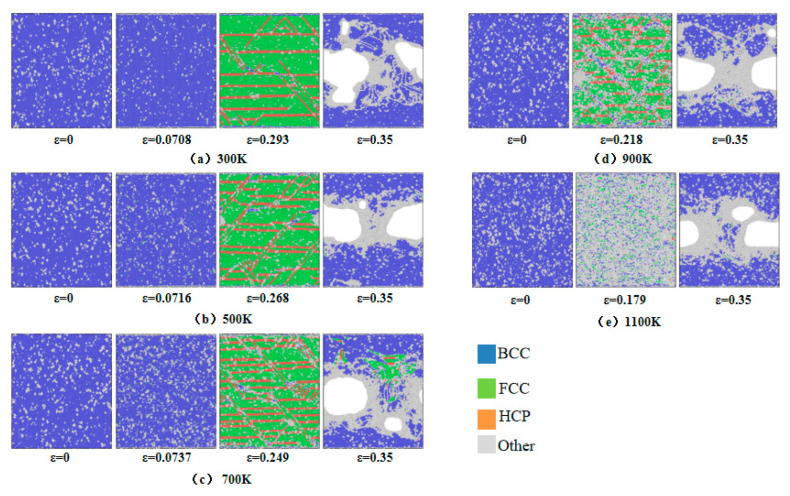
Evolution of crystal structure at 300–1100 K: (**a**) 300K; (**b**) 500 K; (**c**) 700 K; (**d**) 900 K; (**e**) 1100 K.

Radial distribution function analysis indicates that crystal structure transitions occur within the model at the first peak of the stress–strain curve. To investigate microstructural evolution during uniaxial tensile deformation, Common Neighbor Analysis was employed to monitor crystallographic changes. Figure 12 illustrates the microstructural evolution at varying strain levels across 300–1100 K, categorizing atomic configurations into BCC, FCC, HCP, and Other structures. For systems at 300–700 K, five characteristic strain points are analyzed: the first stress peak, void nucleation onset, second stress peak, near-stress minimum, and an additional intermediate point. At 900–1100 K, four points are selected: void nucleation onset, stress peak, near-stress minimum, and a transitional stage. As shown in Figure 12a–e, incipient FCC structures emerge at the first stress peak under 300–700 K conditions, with their population increasing alongside strain accumulation and concurrent HCP phase formation. The transformation from BCC to FCC and HCP structures occurs as a result of thermal fluctuations and reduced energy barriers, leading to the release of localized stress accumulation until the stress level starts to decline. Such structural transitions have been consistently observed in both molecular dynamics simulations [39] and experimental investigations [40]. At void nucleation onset, BCC configurations predominantly convert into FCC, HCP, and Other structures, with the latter concentrating at void nucleation sites. During the second stress peak, increased void nucleation density and volumetric expansion drive BCC phase resurgence. At 900–1100 K, Other structures dominate with diminished FCC/HCP fractions, particularly at 1100 K, where amorphous phases proliferate, signifying temperature-enhanced BCC-to-Other transitions. Post-second peak, rapid void growth correlates with abrupt FCC/HCP depletion and BCC/Other hybrid structure formation. Void coalescence generates microcracks enveloped by amorphous phases, which widen and interconnect under progressive strain, ultimately precipitating material fracture.

**Figure 13:**
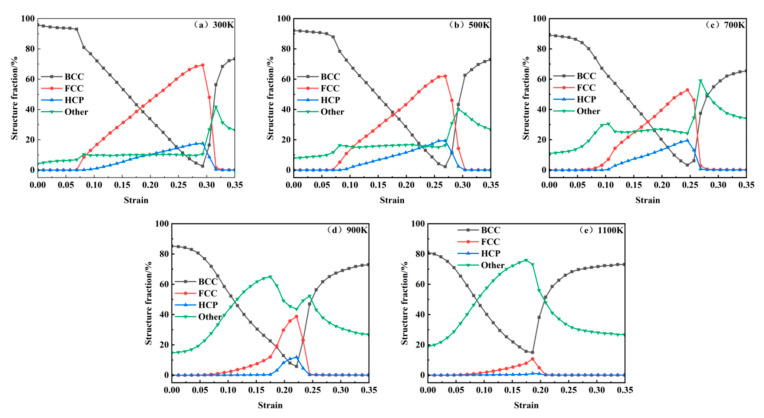
Evolution of different crystal structure types at 300–1100 K.

Figure 12 qualitatively illustrates the evolution of crystallographic phases during tensile deformation. For a quantitative analysis, Figure 13 statistically presents the temperature-dependent proportions of BCC, FCC, and HCP structures as functions of strain across 300 K to 1100 K. At 300 K, the system initially contains a minor fraction of Other structures (4%), attributed to carbon-induced lattice distortions. An increasing temperature elevates the initial proportion of BCC structures from 4% at 300 K to 19% at 1100 K, demonstrating thermally enhanced structural reorganization. Following the first stress peak, FCC configurations emerge and proliferate alongside HCP phase formation. Both phases attain maximum proportions at the second stress peak through strain-driven transformations. The volumetric dominance of FCC structures declines progressively with a rising temperature, decreasing from 70% at 300 K to 62% at 500 K, 53% at 700 K, 39% at 900 K, and ultimately 9% at 1100 K. Concurrently, HCP fractions diminish from 15% at 900 K to 2% at 1100 K, reflecting thermally suppressed phase stability. Accelerated void growth post-second stress peak, as shown in Figure 6, correlates with the abrupt depletion of FCC and HCP phases and the resurgence of BCC configurations. This phase redistribution stems from void coalescence, which initiates crack propagation and redistributes system stress fields. Subsequent atomic rearrangement under localized strain relaxation drives the reconstruction of BCC structures, highlighting the interplay between defect dynamics and crystallographic recovery.

### 3.6. Potential Energy Analysis

**Figure 14:**
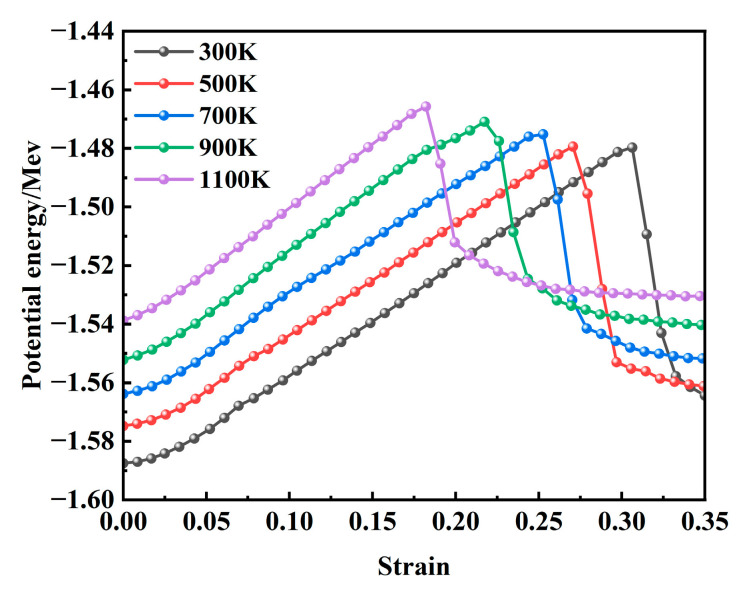
Evolution of system potential energy at 300–1100 K.

Figure 14 presents the variation in internal potential energy as a function of strain during the tensile deformation of the model at various temperatures. At the onset of stretching, the internal potential energy has already reached a stable state due to the prior relaxation process. When correlated with the stress–strain curve shown in Figure 2, it is evident that the potential energy attains its peak value when the stress reaches its maximum under different temperature conditions. During this phase, the potential energy increases gradually with a rising temperature, and the overall trends across different temperatures remain largely consistent. This behavior can be attributed to the fact that elevated temperatures enhance atomic kinetic energy, intensify vibrational frequencies, and lead to increased interatomic distances, resulting in a more disordered atomic configuration, higher free energy, and reduced structural stability. Following the stress maximum, the potential energy rapidly decreases with further strain and subsequently stabilizes. As illustrated in Figure 6, this stage corresponds to the rapid growth of voids, during which a significant number of interatomic bonds are disrupted, leading to a sharp decline in potential energy.

## 4. Conclusions

In this study, the uniaxial dynamic tensile deformation of Fe-C alloy models at temperatures ranging from 300 to 1100 K and a strain rate of 2 × 10^9^ s^−1^ was systematically investigated using molecular dynamics simulations. Through an analysis of stress evolution, void volume fraction, crystal structure transitions, and potential energy variations during the tensile process, the temperature-dependent deformation mechanisms and the mechanisms of crack initiation and propagation in Fe-C alloys were elucidated. The following conclusions were obtained:

(1) When the temperature ranges from 300 to 700 K, the stress–strain curve exhibits two distinct peaks, whereas in the temperature range of 900 to 1100 K, only a single peak is observed. With an increasing temperature, the maximum stress value gradually decreases, ranging from a peak of 14.31 GPa at 300 K to a minimum of 10.7 GPa at 1100 K—a reduction of 25.2%. Additionally, the system’s potential energy reaches equilibrium earlier at higher temperatures. The rapid growth of voids is identified as the primary factor contributing to the sharp decline in stress and potential energy and, furthermore, serves as the fundamental mechanism underlying microcrack initiation.

(2) Void evolution progresses through three stages: incipient growth, accelerated expansion, and stabilized coalescence. Elevated temperatures advance void nucleation onset and amplify system potential energy, with rapid void growth driving stress/potential energy collapse. Dislocations dominate plastic deformation, generating localized pile-ups that induce stress concentration and accelerate crack propagation.

(3) With an increasing temperature, the BCC structure is preferentially transformed into Other structures. Prior to void nucleation, the dominant structural transition involves the conversion of BCC to FCC and HCP configurations. The coalescence of voids induces a redistribution of system stress, which drives atomic rearrangement and subsequently facilitates the re-emergence of the BCC phase.

This study primarily investigated the deformation behavior and crack initiation mechanisms of single-crystal models during the tensile process. To better reflect the microscale deformation behavior and the evolution of crack initiation mechanisms in practical steel, it would be more meaningful to incorporate microstructural features such as grain boundaries and carbides. Furthermore, integrating experimental observations of microstructural changes in the alloy can enable a more comprehensive understanding of the material’s deformation and damage mechanisms.

## Data Availability

The original contributions presented in this study are included in the article. Further inquiries can be directed to the corresponding authors.
